# Correction: Multidimensional assembly using layer-by-layer deposition for synchronized cardiac macro tissues

**DOI:** 10.1039/d0ra90063j

**Published:** 2020-06-04

**Authors:** Yongjun Jang, Da Jung Jung, Seung-Cheol Choi, Do-Sun Lim, Jong-Hoon Kim, Gi Seok Jeong, Jongseong Kim, Yongdoo Park

**Affiliations:** Department of Biomedical Sciences, College of Medicine, Korea University Seoul Korea envokim72@korea.ac.kr ydpark67@korea.ac.kr; Biomedical Engineering Research Center, Asan Institute for Life Sciences, Asan Medical Center Seoul Korea gsjeong@amc.seoul.kr; Departments of Cardiology, Cardiovascular Center, Korea University Anam Hospital Seoul Korea; Department of Biotechnology, College of Life Sciences and Biotechnology, Korea University Seoul Korea

## Abstract

Correction for ‘Multidimensional assembly using layer-by-layer deposition for synchronized cardiac macro tissues’ by Yongjun Jang *et al.*, *RSC Adv.*, 2020, **10**, 18806–18815, DOI: 10.1039/D0RA01577F.

The authors regret that the name of one of the authors (Gi Seok Jeong) was shown incorrectly in the original article. The corrected author list is as shown above.

The Royal Society of Chemistry apologises for these errors and any consequent inconvenience to authors and readers.

## Supplementary Material

